# Combined Effect of Insulin-Like Growth Factor-1 and CC Chemokine Ligand 2 on Angiogenic Events in Endothelial Cells

**DOI:** 10.1371/journal.pone.0121249

**Published:** 2015-04-01

**Authors:** Iana Mayane Mendes Nicácio Viana, Maíra Estanislau Soares de Almeida, Marvin Paulo Lins, Maria Danielma dos Santos Reis, Larissa Fernanda de Araújo Vieira, Salete Smaniotto

**Affiliations:** 1 Laboratory of Cell Biology, Institute of Biology and Health Science, Federal University of Alagoas, Maceió, Alagoas, Brazil; 2 Department of Cell and Developmental Biology, Biomedical Sciences Institute, University of São Paulo, São Paulo, Brazil; University of Bari Medical School, ITALY

## Abstract

Therapeutic angiogenesis may be applied in medical conditions to promote stimulation of angiogenesis. Angiogenesis is a multistep process, which includes endothelial cell proliferation, migration, and tube formation, which is mediated by various angiogenic polypeptides. Thus, studies that elucidate the cellular mechanisms involved in these processes are necessary to develop novel therapeutic strategies. This study investigated the *in vitro* effects of the pro-angiogenic factors, insulin-like growth factor-1 (IGF-1) and/or chemokine (CC motif) ligand 2 (CCL2), on endothelial cells. Flow cytometry analysis showed that IGF-1 and CCL2 treatment did not interfere with IGF-1 receptor (IGF-1R) expression, but CCL2 treatment increased CCL2 receptor (CCR2) expression. Immunofluorescence analysis revealed that the IGF-1/CCL2 combination induced a greater increase in fibronectin deposition, but the treatments did not alter the expression of the fibronectin receptors, CD49e and CD44. The interaction of fibronectin with cytokines demonstrated that IGF-1/CCL2 promoted changes in intermediate F-actin remodeling that may result in increased endothelial cell adhesion and cell migration mediated by fibronectin. Furthermore, IGF-1/CCL2 stimulated endothelial cells, grown on fibronectin, to form capillary-like structures and intercellular lumina with greater luminal area. These data suggest that IGF-1/CCL2 combination and a fibronectin matrix may contribute to the angiogenesis process to stimulate adhesion, migration, and tube formation by endothelial cells as a result of F-actin remodeling.

## Introduction

The endothelium is a monolayer of cells lining the interior of the blood and lymphatic vessels. This cellular layer is attached to the basal membrane and participates in the exchange of materials between blood and tissues. Endothelial cells have essential activities in the control of vascular functions and play an important role in the formation of new blood vessels and restoration of damaged vessels [1, 2]. Endothelial cells release a multitude of biological mediators such as growth factors, vasoactive mediators, coagulation and fibrinolysis proteins, and immune factors. These cells are usually in the quiescent state, reflecting the stability and integrity of the vascular wall [2, 3]. During a series of physiological or pathological processes that involve angiogenesis, such as embryonic development, reproduction, wound repair, and tumor growth [4–6], the resting state changes and endothelial cells become elongated, highly motile, and sensitive to stimulation by growth factors [7].

Insulin-like growth factors (IGFs) and chemokines are major factors that regulate the angiogenesis process [8, 9]. Both circulating and locally produced IGFs are believed to play a role in the regulation of cell proliferation, differentiation, and initiation of apoptosis as well as maintenance and critical regulation of many physiological functions, ranging from longevity to immunity [10, 11]. Insulin-like growth factor-1 (IGF-1) is a single polypeptide with structural homology to insulin-like growth factor-2 (IGF-2) and proinsulin [12, 13]. It is largely produced in the liver under the control of growth hormones [14]. IGF-1 can stimulate endothelial function, differentiation, migration, capillary-like structure formation, and prevention of endothelial dysfunction [15–17].

Chemokine (CC motif) ligand 2 (CCL2), a potent chemotactic factor for monocytes, macrophages, memory T lymphocytes, and natural killer cells, is also a direct modulator of endothelial function [18, 19]. CCL2 can contribute to proliferation, migration, capillary-like structure formation, and endothelial wound repair through the CCL2 receptor (CCR2) [20–24].

Combined effect of IGF-1 or CCL2 with other cytokines in the angiogenesis process has been investigated. IGF-1 is necessary at minimal levels to promote the maximum function of vascular endothelial growth factor (VEGF) and is critical for normal retinal vascular development [8]. Furthermore, CCL2 induced by VEGF or angiotensin-II seems to participate in angiogenesis [25, 26]. IGF-1 and VEGF also exert complementary therapeutic effects in post-infarction heart failure [27]. The goal of therapeutic angiogenesis is to improve perfusion and restore tissue function, leading to a broad range of interventions that allows the growth of new blood vessels to promote neovascularization in healing wounds, diabetic ulcers, peripheral arterial disease, and ischemic tissue [1, 20, 28].

Thus, studies that elucidate the cellular mechanisms mediated by the interaction between pro-angiogenic molecules such as IGF-1 and CCL2 are required for their application in novel therapeutic strategies. However, such research has not been documented in the literature.

In the present study, the effect induced by the IGF-1 and CCL2 combined treatment on endothelial cells, grown on fibronectin (FN), was demonstrated. IGF-1 and/or CCL2 treatment of endothelial cells induced FN deposition, confirming its importance for endothelial cells. Moreover, the rearrangement of the F-actin cytoskeleton promoted by the treatment was associated with endothelial adhesion and migration, leading to the formation of extracellular lumina, which presented increased average area.

## Material and Methods

### Cells and culture conditions

The murine thymic endothelioma cell line (tEnd.1) was provided by Dr. T. C. Barja-Fidalgo (University of Rio de Janeiro, Brazil). tEnd.1, generated by transformation with the polyomavirus middle T oncogene, retains the functional properties of normal endothelium and may represent an invaluable tool for analysis of the immunobiology and heterogeneity of endothelial cells in different tissues [29]. The cells were grown in Roswell Park Memorial Institute (RPMI) 1640 medium supplemented with 10% fetal bovine serum (FBS), 2 mM glutamine, 100 U/mL penicillin, and 100 U/mL streptomycin (all from Invitrogen, Carlsbad, CA, USA) and were cultured at 37°C in a fully humidified atmosphere flushed with 5% CO_2_.

### Proliferation assay

tEnd.1 cells (3 × 10^4^) were seeded in 6-well culture plates in RPMI 1640 complete medium for 16 h for cellular adhesion. After this period, the cells were washed with phosphate buffered saline (PBS) and were treated with recombinant mouse insulin-like growth fator-1 (IGF-1) (Sigma-Aldrich, St Louis, MO, USA) at concentrations of 5, 10, 50, and 100 ng/mL for 8 h. After treatment, cells were counted using a hemocytometer.

### MTT assay for cell viability

tEnd.1 cells (1 × 10^5^) were grown in 96-well plates with RPMI 1640 complete medium for 16 h until cellular adhesion was attained [30]. Cells were then treated with recombinant mouse CCL2/JE/MCP-1 (CCL2) (R&D Systems, Minneapolis, MN, USA) at concentrations of 5, 10, 50, and 100 ng/mL for 24 h. After treatment, cells were incubated with 5 mg/mL of tetrazolium salt (MTT) (Sigma-Aldrich) diluted in RPMI 1640 with 2% FBS. The reduction of MTT by metabolically active cells formed formazan crystals, which were solubilized by the addition of DMSO (Sigma-Aldrich). Spectrophotometer readings were taken at an absorbance of 540 nm (TP-Reader-Thermoplate, Nanshan District, Shenzhen, China).

### Immunocytochemistry

After treatment with IGF-1 and/or CCL2 for 24 h, cells were subjected to an indirect immunofluorescence assay as previously described [31]. Samples were washed with PBS (Sigma-Aldrich), followed by treatment with 1% bovine serum albumin (BSA) (Sigma-Aldrich). The tEnd.1 cells were incubated with a primary anti-FN antibody (rabbit, 1:50; Sigma-Aldrich) for 1 h at room temperature. After additional washes, cells were incubated with the secondary antibody, goat anti-rabbit-FITC conjugated (1:200, Sigma-Aldrich) for 45 min at room temperature. Immunostained samples were analyzed by fluorescence microscopy (Nikon Eclipse 50i; Nikon Instruments Inc., Chicago, IL, USA). Negative controls, in which primary antibodies were replaced by unrelated immunoglobulins or in which the secondary antibody was used alone, did not generate any significant immunolabeling. Quantitative fluorescence analyses were performed by transforming specific staining in pixels and by dividing the total pixel numbers by the area analyzed, obtaining the numbers of pixels/μm^2^.

### Flow cytometry

tEnd.1 cells (10^6^) were treated with IGF-1 and/or CCL2 for 24 h. Next, cells were incubated with appropriate dilutions of the following fluorochrome-labeled monoclonal antibodies: anti-IGF-1R/PerCP, anti-CCR2/FITC (R&D Systems), anti-CD44/PE, and anti-CD49e/PE (BD Pharmingen, San Diego, CA, USA) as previously described [31]. Cells were then evaluated by flow cytometry in a FACS Canto II device (Becton Dickinson, San Jose, CA, USA). Analyses were performed using FACSDiva software (Becton Dickinson).

### Cytoskeleton staining assay

After treatment with IGF-1 and/or CCL2 for 24 h, 2 × 10^3^ cells were seeded in a 24-well plate with round glass coverslips previously coated with 10 μg/mL of FN (Sigma-Aldrich) or 10 μg/mL of BSA and incubated for 16 h. Cells were washed with PBS, fixed, and permeabilized for 5 min with 4% paraformaldehyde in PHEM buffer (60mM PIPES, 2mM HEPES, 10mM EGTA, and 2mM MgCl_2_, Sigma-Aldrich) containing 0.5% Triton X-100 and 5% sucrose (Sigma-Aldrich). Post-fixation was performed for 15 min with the same buffer without Triton X-100. After washing, tEnd.1 cells were stained with phalloidin-Alexa 488 (Molecular Probes, Eugene, OR, USA) for 1 h. Cell spreading was estimated by the area occupied by the cell, using the Image J software (NIH, Bethesda, MD, USA). A confocal inverted microscope (LSM-510, Zeiss, Göttingen, Germany) was used for observation with a 63× objective.

### Cellular adhesion assay

In a 96-well plate previously coated with 0.1% BSA or 10 μg/mL FN, 5 × 10^4^ cells treated with IGF-1 and/or CCL2 for 24 h were added in each well to measure the cellular adhesion. Non-adherent cells were washed away 1 h later. Adherent cells were fixed with formaldehyde and stained with crystal violet (Sigma-Aldrich). Spectrophotometer readings were taken at an absorbance of 540 nm [32].

### Endothelial migration assay

Migration of tEnd.1 was assessed using a transwell system, which consists of inserts with polycarbonate membranes having a diameter of 10 mm and a pore size of 8.0 μm (Corning Costar, Cambridge, MA, USA). A suspension of 2 × 10^5^ tEnd.1 cells was added to the upper chamber of inserts previously coated with 0.1% BSA or 10 μg/mL FN, and IGF-1 and/or CCL2 were used as chemotactic factors. After 6 h of migration, migrant cells in the bottom chamber were fixed, stained, and counted using methods previously described [33].

### Cellular morphological analysis

tEnd.1 cells (5 × 10^3^) were cultured on round slides in 24-well plates. The culture was treated with IGF-1 and/or CCL2 for 24 h in RPMI 1640 with 2% FBS. Cells were then fixed with methanol and stained with Giemsa. Subsequently, the coverslips were mounted on permanent slides and analyzed by light microscopy. Photographs were obtained by using a Nikon DS-Ri1 camera coupled to a Nikon Eclipse 50i microscope (Nikon Instruments Inc.).

### Capillary-like network formation assay

The ability of tEnd.1 cells to form capillary-like structures was evaluated on surfaces coated with 0.1% BSA or 10 μg/mL FN, as described previously [34] with some modifications. The FN coating was prepared on round glass coverslips in a 24-well plate and the cells were plated. On the fourth day, the culture medium was changed and treatment was renewed. On the eighth day, cells were fixed and stained, and the coverslips were mounted on permanent slides and analyzed by light microscopy. Luminal area and the formation of capillary-like structures were measured by DP2-BSW software (version: Olympus Soft Imaging Solution GmbH, Munster, Germany).

### Statistical analysis

The data obtained were analyzed using one-way or two-way ANOVA, followed by Bonferroni’s post-test. The values are presented as the mean ± standard error of the mean (SEM) and considered significant when p ≤ 0.05.

## Results

### CCL2 increased CCR2 expression in endothelial cells

**Fig 1:**
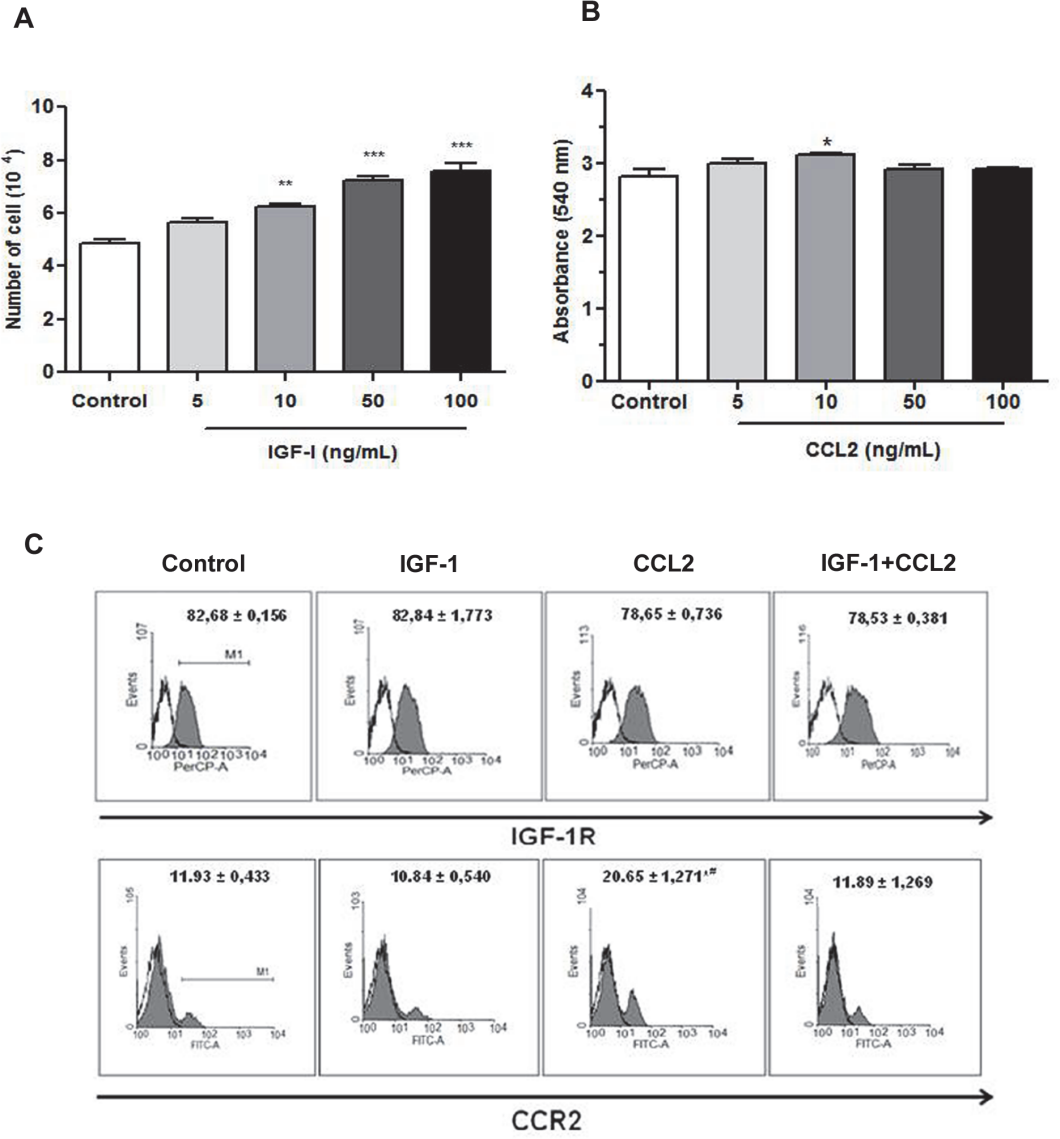
IGF-1 or CCL2 stimulated endothelial cell viability. tEnd.1 cells were treated with IGF-1 (A) or CCL2 (B) at concentrations of 5, 10, 50, or 100 ng/mL, and cell viability was determined by cell counting using a hemocytometer or MTT assay, respectively. (C) Flow cytometry results are presented as histograms of the average percentage of cells that expressed IGF-1R and CCR2 receptors (gray) and immunoglobulin control. Values and bars are represented as the mean ± SEM (n = 4/group). Results were analyzed by one-way ANOVA followed by Bonferroni’s post-test. Significant values compared to the control group: p < 0.05 (*) or p < 0.0001(***); significant value compared to control group and the other treatments: p < 0.0001 (#).

IGF-1 and CCL2 concentrations were determined on the basis of the murine thymic endothelioma cell line (tEnd.1) proliferation and cell viability. A significant increase in cell number was observed in presence of IGF-1 at 10, 50, and 100 ng/mL (Fig. 1A). Treatment with 10 ng/mL of CCL2 significantly stimulated endothelial cell viability (Fig. 1B). The influence of IGF-1 and CCL2 on the biological properties of endothelial cells is mediated via their respective receptors. The effect of IGF-1 and/or CCL2 on the expression of their respective receptors was analyzed by flow cytometry. tEnd.1 cells expressed both receptors. A high percentage of cells expressed IGF-1R (82% ± 0.156%) and a lower percentage of cells expressed CCR2 (11% ± 0.433%) (Fig. 1C). IGF-1 and/or CCL2 treatment did not interfere with the percentage of cells that expressed IGF-1R. However, the percentage of cells that expressed CCR2 increased significantly (73%) after treatment with CCL2 alone than that of the untreated control, IGF-1, and combined IGF-1/CCL2 treated cells (Fig. 1C).

### IGF-1/CCL2 combination augmented fibronectin deposition by tEnd.1 cells

**Fig 2:**
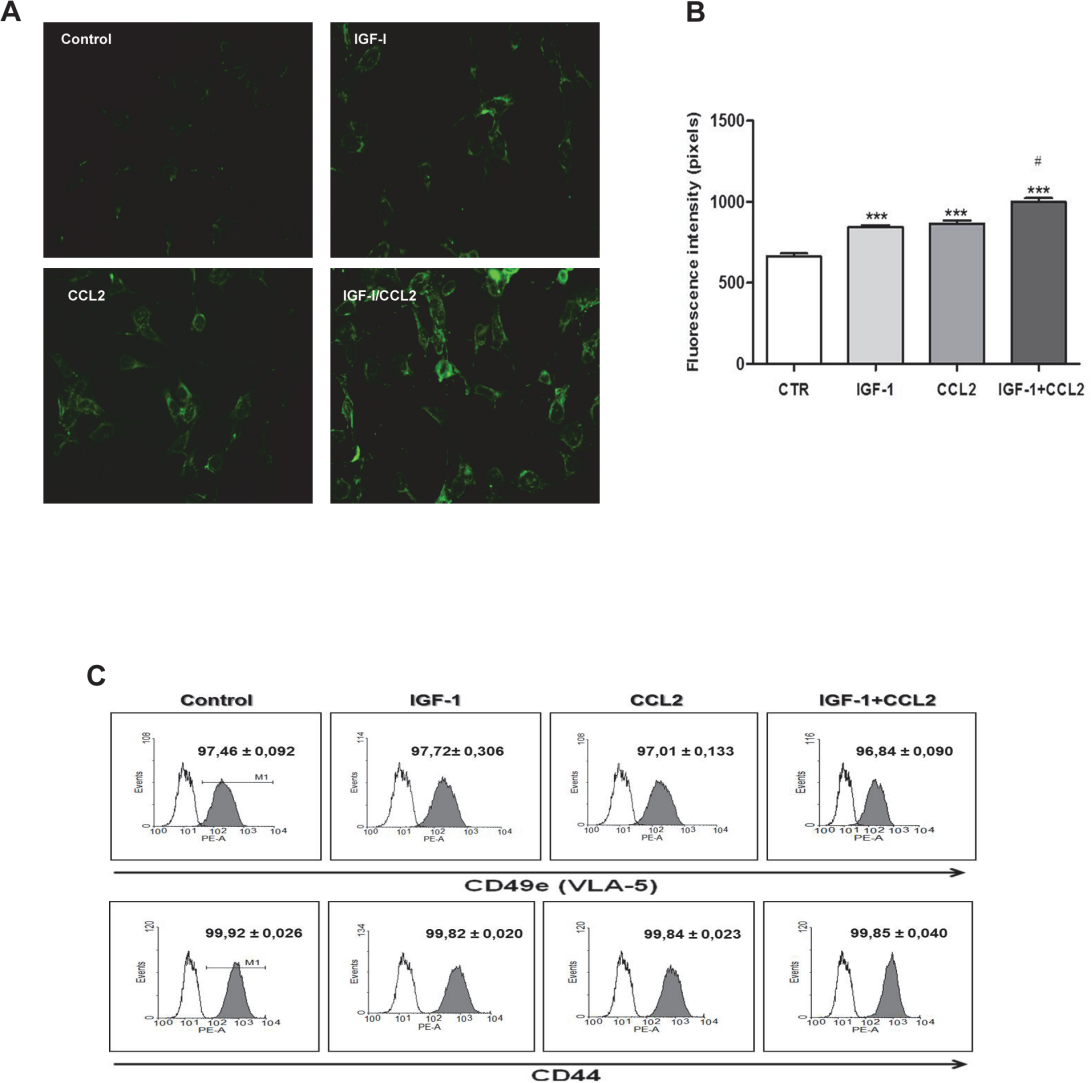
IGF-1 and/or CCL2 augmented fibronectin deposition in tEnd.1 cells. tEnd.1 cells were treated with IGF-1 (100 ng/mL), CCL2 (10 ng/mL), or a combination of both for 24 h and analyzed by fluorescence microscopy. (**A**) Photomicrographs show the expression of FN ascertained by immunofluorescence and fluorescence microscopy analysis. Magnification: 400× (**B**) Bars correspond to the quantitative analysis of FN expression in tEnd.1 cells in selected microscopic fields (n = 5/group). The results are expressed in pixels/μm^2^. (**C**) Flow cytometry results are presented as histograms of the average percentage of cells that expressed CD49e/VLA-5 and CD44 receptors for FN (gray) and immunoglobulin control. Values and bars are represented as the mean ± SEM (n = 5/group). Results were analyzed by one-way ANOVA followed by Bonferroni’s post-test. Significant values compared to control group: p < 0.0001 (***); significant values compared to control group and single treatments: p < 0.0001 (#).

tEnd.1 cells expressed IGF-1 and CCL2 receptors and could induce matrix deposition. As a major structural component of resistant vessels, the extracellular matrix (ECM) plays a substantial role in the maintenance of vessel integrity [35]. Therefore, endothelial cell properties are not only regulated by cytokines, but also in conjunction with ECM molecules. The effect of IGF-1 and/or CCL2 on ECM deposition was determined by FN immunostaining. Qualitative analysis showed that IGF-1 and/or CCL2 treatment increased FN deposition (Fig. 2A). This was confirmed by quantitative fluorescence intensity, which demonstrated a significant increase in FN accumulation. In addition, FN deposition after IGF-1/CCL2 combination treatment was significantly higher than in the control and single treatments (Fig. 2B). The interaction of FN with cell surface receptors, usually through binding of the α5β1 integrin receptor, induces FN activation [36]. Therefore, the effect of IGF-1 and/or CCL2 on the expression of FN receptors CD49e (α5/VLA5) and CD44 in tEnd.1 cells was analyzed by flow cytometry. The results indicated that a high percentage of cells expressed CD49e (97% ± 0.092%) and CD44 (99% ± 0.026%) (Fig. 2C). However, IGF-1 and/or CCL2 treatment did not alter the percentage of cells expressing these receptors as compared to the untreated control cells (Fig. 2C).

### IGF-1/CCL2 combination promoted F-actin cytoskeleton organization

**Fig 3:**
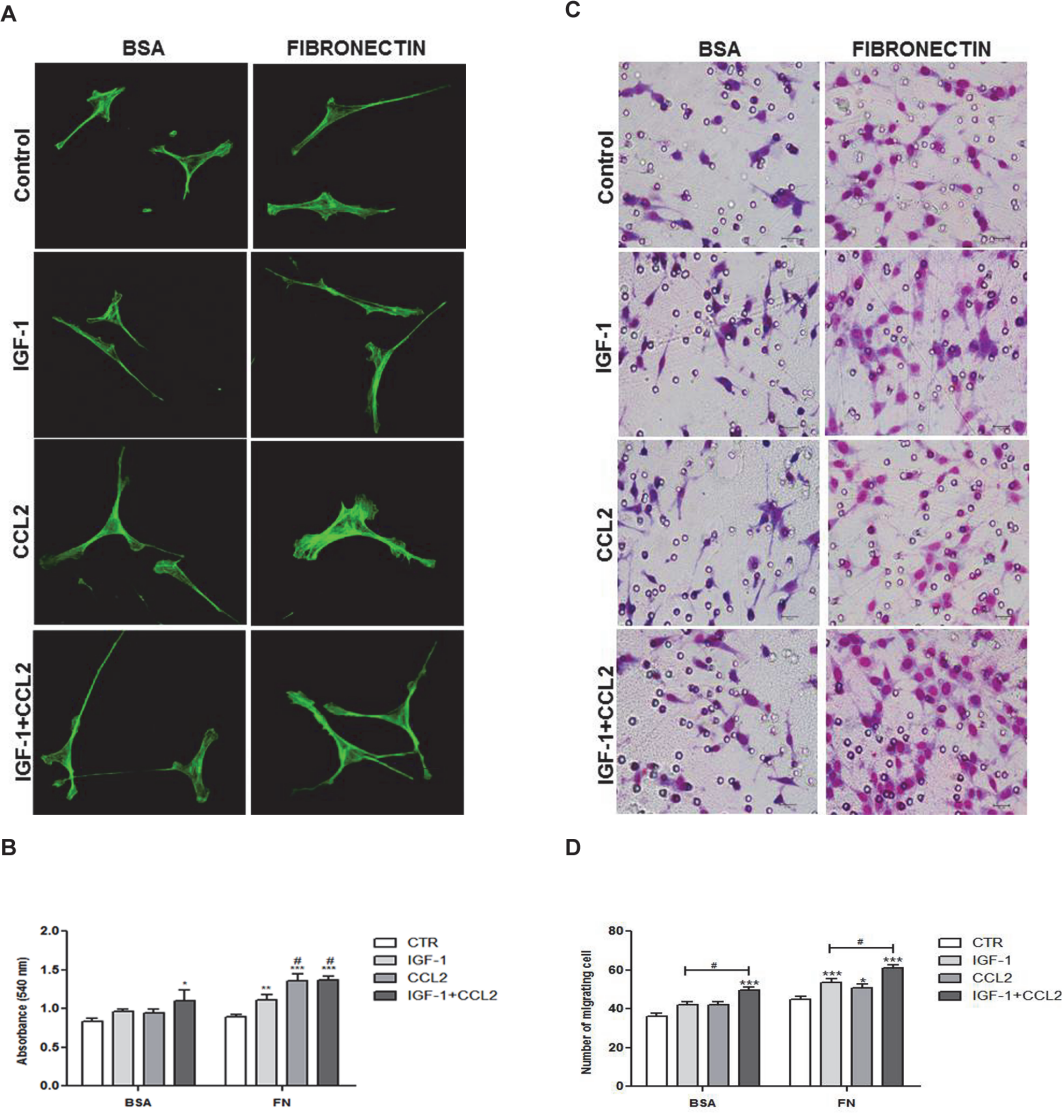
IGF-1 and/or CCL2 increased adhesion and migration of tEnd.1 cells. **(A)** tEnd.1 cells treated with IGF-1 (100 ng/mL), CCL2 (10 ng/mL), or a combination of both for 24 h on BSA or FN coating were stained with Alexa 488-phalloidin and analyzed by confocal microscopy with a 63× objective. (**B**) tEnd.1 cells were allowed to adhere on BSA- or FN-coated surfaces for 1 h after stimulation with IGF-1, CCL2, or IGF-1/CCL2 for 24 h. (**C**) tEnd.1 cells were allowed to migrate through transwell chambers coated with BSA or FN after chemotactic stimulation with IGF-1, CCL2, or IGF-1/CCL2 for 6 h. Photomicrographs demonstrate cells invading through the transwell membrane. Giemsa staining. Scale bar = 10 μm. (**D**) Bars represent the number of migrating cells in a transwell system. Data are represented as mean ± SEM (n = 5/group). Results were analyzed by two-way ANOVA followed by Bonferroni’s post-test. Significant values compared to control group: p < 0.05 (*), p < 0.01 (**), or p < 0.0001 (***); significant values compared to control group and the IGF-1 treatment: p < 0.05 (#); and significant values compared to control group and single treatments: p < 0.01 (+).

To investigate whether cytokines and FN interact to stimulate actin cytoskeleton organization, the effect of IGF-1 and/or CCL2 on F-actin was determined on BSA- or FN-coated surfaces, followed by direct staining with phalloidin. Ours results showed that F-actin cytoskeleton on FN matrix was more spreading (2.2×) than that of the cells grown on BSA without treatment. Moreover, IGF/CCL2 treatment of cells grown on the FN matrix increased the cell number (1.6×) and induced larger (2.6×) lamellipodia than those of the cells grown on BSA coating (Fig. 3A). IGF-1 treatment resulted in a more elongated cytoskeleton, while IGF-1/CCL2 combination treatment increased number and area of lamellipodia.

### IGF-1/CCL2 combination increased tEnd.1 cell adhesion and promoted migration

Intermediate levels of cytoskeletal linkage proteins are associated with maximal migration [37]. Thus, we evaluated whether the cytoskeletal organization promoted by the IGF-1/CCL2 combination treatment of tEnd.1 cells grown on the FN matrix would affect their adhesion and migration. The adhesion capacity was determined through 1 h adherence on BSA or FN-coating surfaces after treatment with IGF-1 and/or CCL2. tEnd.1 cells presented a lower adherence to the BSA-coated surface and only the IGF-1/CCL2 combination increased tEnd.1 cell adhesion (Fig. 3B). However, all treatments significantly increased the adhesion of tEnd.1 cells on the FN-coated surface. Furthermore, CCL2 and the IGF-1/CCL2 combination significantly increased adhesion as compared to the control and IGF-1 treatment alone (Fig. 3B). To assess the effect of IGF-1/CCL2 combination on cell motility *in vitro*, transwell chambers were coated with BSA or FN and cell migration was determined by the number of migrating cells that adhered to the bottom of the transwell membrane after chemotactic IGF-1 and/or CCL2 treatment (Fig. 3C). The chemotactic response of tEnd.1 cells was significant only when cells were stimulated by the IGF-1/CCL2 combination on the BSA-coated surface and this response was higher than that of the single treatments (Fig. 3D). However, tEnd.1 cells significantly migrated after stimulation with all treatments on the FN-coated surface as compared to the control (Fig. 3D). In addition, cells stimulated with the IGF-1/CCL2 combination showed a peak of migration that was statistically significant compared to the control group and single treatments, showing a synergistic effect of IGF-1 and CCL2 on cell migration (Fig. 3D).

### IGF-1/CCL2 stimulated intra- and intercellular lumen formation by tEnd.1 cells

**Fig 4:**
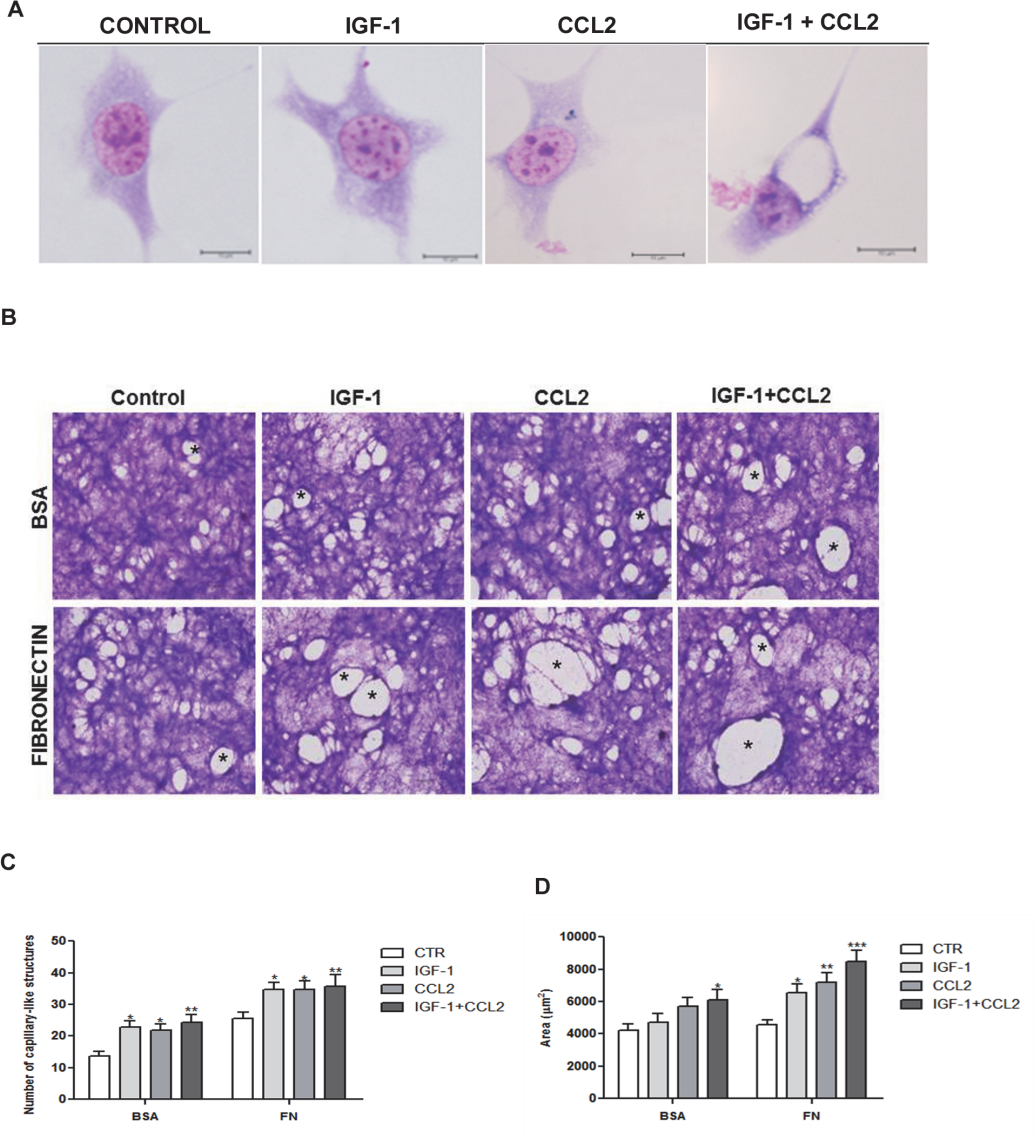
IGF-1 and CCL2 stimulated tEnd.1 cell morphology and tube formation. (**A**) tEnd.1 cells were treated with IGF-1 (100 ng/mL), CCL2 (10 ng/mL), or a combination of both for 24 h and analyzed by optical microscopy. Photomicrographs show intracellular lumina in tEnd.1 cells, indicated by arrows. Giemsa staining. Scale bar = 10 μm. (**B**) tEnd.1 cells were treated with IGF-1, CCL2, or IGF-1/CCL2 for 8 days on BSA or FN coating and analyzed by optical microscopy. Photomicrographs demonstrate capillary-like structures, indicated by asterisks. Giemsa staining. Scale bar = 10 μm. (**C**) Number of capillary-like structures. (**D**) Luminal area of capillary-like structures. Bars represent the mean ± SEM (n = 6/group). Results were analyzed by two-way ANOVA followed by Bonferroni’s post-test. Significant compared with control, p < 0.05 (*), p < 0.01 (**), or p < 0.001 (***).

Because the IGF-1/CCL2 combination stimulated cell migration, we investigated whether cytokines could modulate tubulogenesis *in vitro*. The morphological analysis of tEnd.1 cells demonstrated that the combined IGF-1/CCL2 treatment for 24 h promoted intracellular lumen formation in the absence of ECM (Fig. 4A). When cells remained in culture for a longer period (8 days) on BSA- or FN-coated surfaces, they showed the ability to form more complex structures similar to the capillaries (Fig. 4B). The number of capillary-like structures formed after treatment with IGF-1 and/or CCL2 was statistically higher than the untreated cells grown on both BSA- or FN-coated surfaces (Fig. 4C). The luminal area of capillary-like structures was measured and only cells treated with the IGF-1/CCL2 combination showed a significant increase in luminal area in cells grown on BSA-coated slides, whereas luminal area was significantly increased by all treatments in cells grown on FN-coated slides (Fig. 4D).

## Discussion

Therapeutic angiogenesis refers to the beneficial application of angiogenesis stimulation in medicine. This therapy may be achieved by administering pro-angiogenic polypeptides and can be employed in wound healing, fracture repair, reconstructive surgery, and collateral vessel formation [38]. Furthermore, the combination of growth factors could have important implications for the treatment of severe arterial insufficiency in patients with diseases that are not amenable to direct revascularization [39]. This study demonstrated that a combination of IGF-1 and CCL2 could induce the migration of tEnd.1 cells *in vitro*, contributing to tubulogenesis and lumen formation with greater average area on a FN matrix.

As previously observed, in human umbilical vein endothelial cells [20, 21, 40], tEnd.1 cells expressed IGF-1 and CCL2 receptors. However, tEnd.1 cells express more IGF-1 receptors than CCR2. Low CCR2 expression in tEnd.1 cells was also demonstrated in bEnd.3 cells in which the level of expression of CCR2 mRNA under resting conditions might be the result of specific *in vitro* conditions, which have been shown to be critical for the downregulation of CCR2 in monocytes or macrophages [41, 23]. CCL2 alone significantly stimulated CCR2 expression, while IGF-1/CCL2 treatment did not affect CCR2 expression. Yet, it is conceivable that the mechanisms controlling the expression of this molecule in tEnd.1 cells are different depending on the stimulus. Thus, more studies on intracellular signaling are necessary to explain the mechanisms of interaction between IGF-1/CCL2 in endothelial cells.

The interaction between cells and extracellular components is essential in determining cellular behaviors in tissues [42]. Several ECM compounds act on the endothelial function together with cytokines present in the matrix. The IGF-1 and/or CCL2 effect on increased FN deposition was also shown in bEnd.3 cells and epithelial cells [23, 43]. However, IGF-I did not affect FN production in human corneal epithelial cells (HCECs) [44]. Differences in FN expression after IGF-1 treatment could be justified by the accumulation and/or binding of FN molecules to the FN receptors on the cell surface [45]. Considering that the expression of FN receptors was not affected, it will be useful to evaluate the expression of integrin subunit αv, which, through integrin αvβ3, promotes recycling to focal contacts required for persistent migration [46, 47] and tyrosine phosphorylation of focal adhesion kinase, which plays an important role in the regulation of cell morphology and in promoting cell migration events [48, 49]. However, previous studies demonstrated that IGF-1 or CCL2 treatment upregulated the expression of β1 integrin in HCECs and of α5, αv, and β3 in bEnd.3 cells [43, 23], but IGF-1 did not upregulate α3 expression in HCECs [43].

Integrin–ligand binding triggers actin cytoskeleton organization at specific sites on the surface membrane to facilitate cell movement or maintain tissue stability [50]. The interaction between the ECM and IGF-1 or CCL2 on the cytoskeleton of tEnd.1 cells cultured on a FN-rich matrix was similar to that observed in previous studies in epithelial cells and bEnd.3 cells [51]. The F-actin reorganization promoted by IGF-1/CCL2 association induced more changes in tEnd.1 cells, stimulating active cytoskeleton reorganization and elongated configuration, to stimulate the formation of microspikes, i.e., very short filopodia almost completely embedded in the cell cortex or leading edge [52]. This F-actin remodeling likely affected the adhesion and had an effect on tEnd.1 cell migration on the FN matrix. A significant peak of migration was observed when tEnd.1 cells were treated with IGF and CCL2, which probably means a change in the cell behavior. The maximal migration may be justified by active changes during cytoskeleton remodeling because lamellipodia and filopodia are essential for cell motility and substrate adhesion [53]. In addition, elongated cytoskeleton configuration mimics the plane configuration, which increases sensitivity to specific growth factors during vasodilatation [54, 4].

Angiogenesis is defined as the formation of new blood vessels from pre-existing vessels via sprouting [55]. Sprouting endothelial cells assemble into solid cords, which undergo tubulogenesis to form vessels with a central lumen [56, 57]. In this study, we showed that IGF-1/CCL2 combination treatment of tEnd.1 cells led to intracellular lumina and coalescent vacuoles, driving vascular lumen formation. Previous studies have demonstrated that intracellular and intercellular fusion of endothelial vacuoles drives vascular lumen formation [58, 59]. IGF-1 and CCL2 also possess the ability to incorporate vascular networks [16, 23]. However, the luminal area of capillary-like structures on FN matrix was accentuated by the IGF-1/CCL2 combination treatment, as previously described for the combination of VEGF and basic fibroblast growth factor on angiogenesis. The combination of growth factors stimulated significantly greater and more rapid augmentation of collateral circulation, resulting in superior hemodynamic improvement [39]. In addition, when used together, IGF-1 and VEGF exerted complementary therapeutic effects in post-infarction heart failure [27].

Potential angiogenic activity of IGF-1 associated with CCL2 in the presence of FN matrix indicates new properties for pro-angiogenic peptides employed in therapeutic angiogenesis. This underscores the importance of further studies to elucidate the possible mechanisms involved in the combined effect of IGF-1 and CCL2 on endothelial cells.
